# From detections to demography: methodological challenges and opportunities in survival estimation from automated telemetry networks

**DOI:** 10.1186/s40462-026-00711-4

**Published:** 2026-11-05

**Authors:** Scott W. Yanco, Clark Rushing, Bryant C. Dossman, Matthew B. Ogburn, Michael T. Hallworth, Nathan W. Cooper

**Affiliations:** 1 Migratory Bird Center, Smithsonian’s National Zoo and Conservation Biology Institute, Washington, DC 20008 USA; 2 Warnell School of Forestry and Natural Resources, University of Georgia, Athens, GA 30602 USA; 3 The Earth Commons Institute, Department of Biology, Georgetown University, Washington, DC 20057 USA; 4 Fisheries Conservation Lab, Smithsonian Environmental Research Center, Edgewater, MD 21037 USA; 5 Vermont Center for Ecostudies, White River Junction, VT USA

## Abstract

Survival is a fundamental demographic process influencing individual fitness and population dynamics. Automated telemetry systems offer unprecedented opportunities to estimate survival of highly mobile organisms across broad spatial and temporal scales. However, the structure of these tracking networks creates systematic biases that render standard survival models inadequate. Receiver stations are distributed unevenly across landscapes and over time, meaning that individuals taking different movement paths encounter vastly different opportunities for detection. This spatial clustering also creates problematic dependencies: animals detected in receiver-dense areas are more likely to be detected again, while those in sparse areas may disappear from view despite being alive. These observation biases can easily be mistaken for genuine differences in survival, leading to incorrect ecological or conservation inferences. Here, we review the unique challenges posed by automated telemetry data for survival estimation, highlight existing methodological solutions including spatially explicit approaches and modified capture-mark-recapture frameworks, and provide recommendations for advancing this critical application of wildlife tracking networks.

**Supplementary information:**

The online version contains supplementary material available at https://doi.org/10.1186/s40462-026-00711-4.

## Introduction

Determining where, when, and why, organisms die is critical to advancing both basic and applied questions in ecology, evolution, management, and conservation [1–4]. The vast literature on methods for estimating survival, including hundreds of articles and multiple textbooks, reflects the fundamental importance of this demographic process. Despite the wide array of methods available for estimating survival, researchers have only scratched the surface of describing survival rates of most animal species or quantifying the key variables that influence survival, including environmental processes, seasonality, or individual traits [4]. More practically, biodiversity loss is accelerating globally [5, 6], yet the demographic mechanisms driving those losses remain poorly understood for most species, hampering effective conservation action [3, 7–9]. Whether for basic or applied purposes, quantifying survival rates and the processes governing them remains a key challenge for ecologists.

A major obstacle to understanding survival patterns has been the inability to observe small, highly-mobile organisms such as songbirds, bats, and insects as well as many aquatic organisms, over large areas and with sufficient resolution to allow survival estimation. Historically, survival estimates for wildlife have relied on capture-mark-recapture (CMR) methods, which require marked individuals to be physically recaptured or visually re-sighted [10, 11]. Although CMR methods are well developed and widely used, the need to recapture or resight marked individuals generally limits the use of these methods to species and periods of the annual cycle with relatively high site fidelity or research effort [12]. Furthermore, because recapture or re-sight probabilities are often low, CMR studies generally require substantial effort over multiple years to produce reliable survival estimates. Traditional CMR frameworks have remained largely inadequate for estimating survival of species with low site fidelity or during life stages characterized by extensive movement where recapture and resight rates are low, such as migration, natal dispersal, or post-breeding periods [3].

**Fig. 1:**
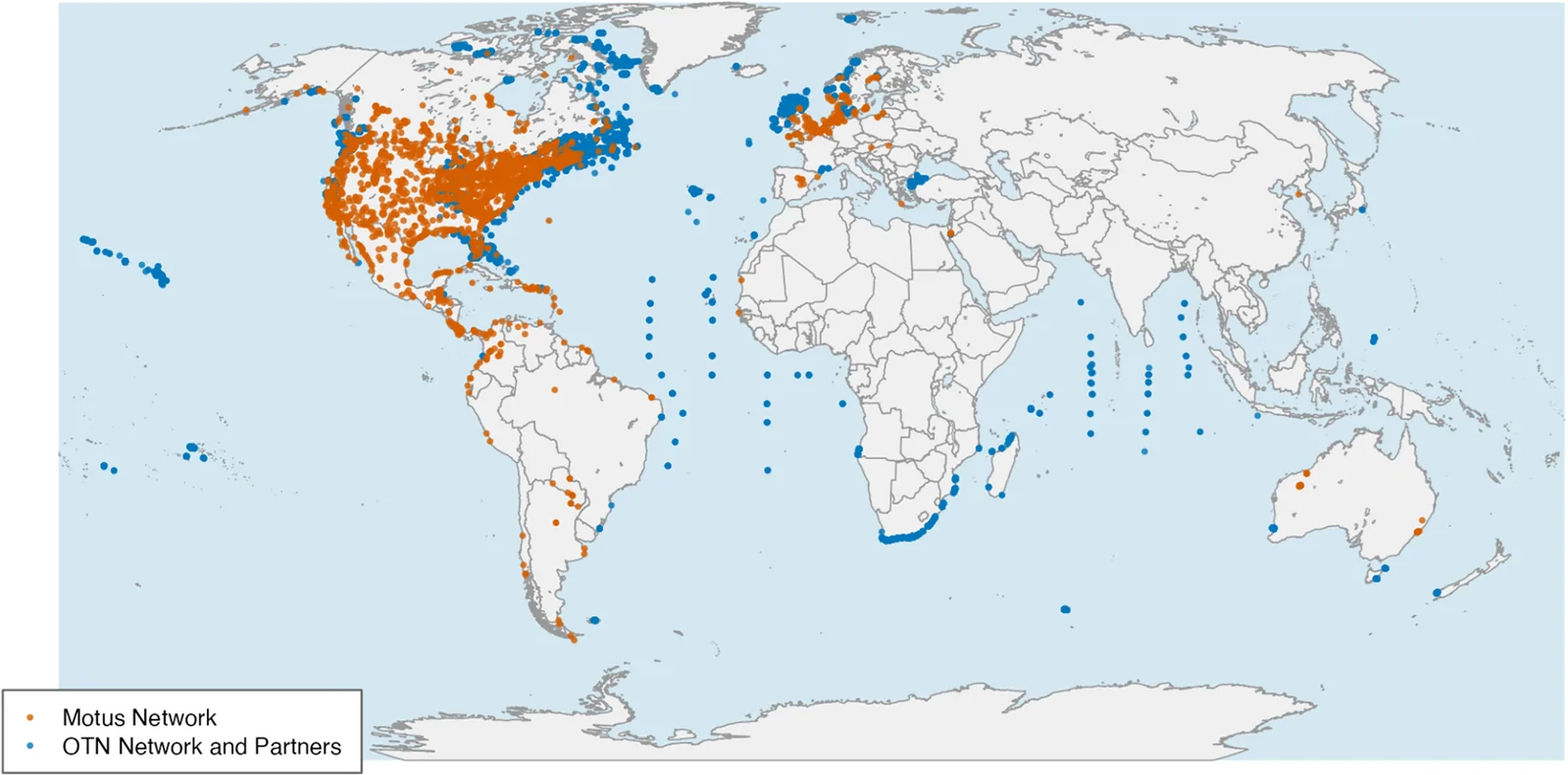
Global coverage of major automated telemetry networks

In recent years, the growth of large, coordinated networks of automated telemetry receivers has created new opportunities to estimate survival at larger spatial scales and during periods of extensive movement, with potential to improve our understanding of spatial and temporal variation in mortality risk for hundreds of highly-mobile, small-bodied, and aquatic species [7, 13–18]. In marine and freshwater systems, over 20,000 acoustic receivers have been deployed by research partnerships across the globe, tracking hundreds of species and generating over 2 billion detections of 457 species (approximately 150,000 individuals) managed by the Ocean Tracking Network (OTN; Fig. 1) [19]. On land, the Motus Wildlife Tracking System (Motus), is a network of approximately 2,300 automated receiver stations (hereafter stations) in 34 countries on six continents (Fig. 1) [13]. Motus has been used to track over 61,000 individual animals from 470 species, including small, highly mobile organisms such as songbirds, bats, and insects (motus.org). Together these networks have provided insights into animal behavior [20–25], ecology [26–29], evolution [30–33], and conservation [34–38] across unprecedented spatial and temporal scales. Yet despite this promise, realizing the potential of these systems to contribute to survival estimation requires recognizing that these data fundamentally differ from traditional mark-recapture datasets.

In many respects, automated telemetry data resemble traditional CMR data: organisms are marked at known locations and times, and subsequent detections provide information about survival and movement. By automating detection and providing spatial coverage across large landscapes, these networks can overcome key limitations of traditional CMR methods. However, as with all CMR data, detections from automated telemetry networks reflect both ecological processes (survival and movement) and observation processes (receiver arrangement, detection range, and network structure). These observation processes are generally more complex than those of many traditional CMR studies, presenting unique challenges for survival estimation.

Automated telemetry has the potential to revolutionize our understanding of survival and movement of many species, but accurate inferences about ecological processes from these networks require careful study design and thoughtful development of analytical methods tailored to the particularities of the data they produce. Here, we outline several key challenges to estimating survival from automated telemetry data and call for additional work to develop statistical methods suited to these data. By outlining the unique properties of automated telemetry data that make survival estimation challenging and highlighting existing or new modeling frameworks that may be useful for advancing analysis of these data, we hope to guide future research in directions that will help unlock the potential of automated telemetry systems to close research gaps and support conservation efforts.

### A primer on survival estimation

Before discussing the challenges of estimating survival using automated telemetry data, we first review the basic principles of capture-mark-recapture (CMR) survival modeling using the Cormack-Jolly-Seber (CJS) model [39–41]. We focus on the CJS model both because it is widely used in wildlife ecology and because its assumptions are well-documented, though the concepts outlined in this paper broadly apply to most survival models that use CMR data (e.g., [42–46]). For more comprehensive treatments of CMR theory and model variants, we refer readers to [10, 47].

The CJS model estimates apparent survival probability (typically denoted ϕ) of individuals between discrete sampling occasions, while accounting for imperfect detection of marked individuals. The term “apparent survival” is used to acknowledge that the survival probability estimated by the CJS model is the confounded combination of true survival and availability, defined as a non-zero probability of being detected, conditional on being alive. CJS models assume that dead individuals cannot be detected or captured, though live individuals can become unavailable (i.e., *p* = 0) for a variety of reasons, including leaving the study area, tag loss, or behavioral changes that prevent capture/detection. Because the model cannot distinguish between dead individuals and unavailable individuals, researchers acknowledge that apparent survival underestimates true survival probability [10].

Although apparent survival is, by definition, an underestimate of true survival when animals are unavailable, obtaining unbiased estimates of apparent survival using the basic CJS model still requires meeting several fundamental assumptions [10]:

1. Every marked animal present in the population at sampling period *i* has the same probability of being captured or re-sighted (*p*).
2. Every marked animal present in the population at sampling period *i* has the same probability of survival until sampling period *i + 1* (ϕ).
3. Marks are neither lost, overlooked, nor recorded incorrectly.
4. Sampling occasions are instantaneous or, in practice, at least very brief relative to intervals between occasions.
5. An individual’s availability for detection at occasion *i* is independent of whether it was available at occasion *i*-1 (i.e., emigration from the study area is permanent).
6. The fate of each animal with respect to capture and survival probability is independent of the fate of any other animal.

As with any model, these assumptions can be relaxed by adding complexity to the model structure and there is a rich literature describing how the CJS model can be modified to accommodate violations of each of these assumptions [42, 48–50]. For example, assumptions 1 and 2 can be relaxed by building individual- or group- varying linear predictors of *p* or ϕ that include relevant covariates or hierarchical structure [10]. Similarly, many common violations of assumption 5 (e.g., trap “happy” or “shy” animals) can be accommodated by conditioning capture probability on an individual’s previous capture history, though modeling among-individual heterogeneity in these behavioral effects often require modified sampling designs (e.g., robust design [48]).

In the following sections, we describe how data from automated telemetry networks violate the assumptions of CJS and other CMR models and also how existing approaches to relaxing these assumptions can provide some solutions for improving survival estimates from these systems.

### Estimating survival with automated telemetry data

To date, automated telemetry has primarily been used to estimate survival at small spatial and temporal scales. In birds, for instance, researchers have used automated radio telemetry, often in combination with traditional handheld telemetry, to estimate survival using known fate models of resident species over several months [51] and in migratory species at relatively small spatial scales and short-duration periods of the annual cycle (i.e., post-fledging, stopover in birds) with low rates of movement [52–54]. Similarly, aquatic ecologists have used automated acoustic telemetry to estimate survival at relatively small spatial scales and over short time periods in bays and open water [55, 56], but also across large, structurally simple river and sound systems [57–62].

In this paper, we describe how estimating survival with automated telemetry at all spatial scales can introduce several possible violations of model assumptions, though these problems are exacerbated at large spatial scales. The most common (and most problematic) are associated with the spatially and temporally irregular natures of the receiver arrays, as well as the difficulty in accounting for tag failure and loss. All of these violations of model assumptions can lead to biased estimates of survival (Table 1). We also include a worked example, using simulated data, demonstrating how the most significant of these problems leads to inferential biases (Supplementary Material).

**Table 1:** Assumptions of CJS models with problems, and potential solutions, specific to automated telemetry

	Assumption	Automated telemetry-specific problems	Potential solutions	Key Citations
#1	Homogeneous probability of detection (*p)* among individuals.	Among-individual differences in movements through an irregular array causes heterogeneity in detection probability.	Account for among individual variance in detection probability directly using movement models to estimate route-specific availability for detection.	[50, 63–65]
#2	Every marked animal present in the population at sampling period *i* has the same probability of survival (ϕ) until sampling period *i + 1*.	Among individual differences in movements coincide with heterogeneity in survival probabilities that cannot be distinguished from variation in detection caused by irregular array, which causes differential bias in apparent survival (ϕ).	Account for among individual variance in detection probability directly using movement models to estimate route-specific availability for detection.	[50, 63–65]
#3	Marks are neither lost, overlooked, nor recorded incorrectly.	a) Tag failure is indistinguishable from non-detection by other processes and can thus bias estimation of apparent survival (ϕ). b) False positive detections caused by radio interference.	a) Relatively unexplored. Model tag failure as a time-varying process using known battery life curves; right-censor individuals at expected tag lifespan; utilize additional data sources to inform tag loss or survival estimates (e.g., double tagging via leg bands), multi-state approaches to model tag status directly. b) Robust outlier detection and filtering.	[49, 66–69]
#4	Sampling occasions are instantaneous, or in practice, at least very brief relative to intervals between occasions.	Automated telemetry provides a continuous detection process; detections can span arbitrarily defined sample “occasions” and post-hoc aggregation schemes could produce different survival estimates.	Define occasions carefully relative to the biology of the study system; use time-to-event or continuous-time survival models rather than forcing data into discrete occasions; conduct sensitivity analyses across aggregation schemes.	[49, 70, 71]
#5	An individual’s availability for detection at occasion *i* is independent of whether it was available at occasion *i*-1 (i.e., emigration from the study area is either permanent or temporally random)	Clustered receiver arrays create autocorrelation in availability, creating Markovian availability and violating the assumption of random temporary emigration.	Robust design to explicitly estimate availability based on prior state; spatially explicit CJS models that account for array geometry.	[17, 48, 50, 64, 71, 72]
#6	The fate of each animal with respect to capture and survival probability is independent of the fate of any other animal.	Likely a minor concern. Individuals from the same tagging cohort or capture site may share similar migratory routes and thus encounter the same portions of the receiver array, creating non-independence in both detection and apparent survival through shared spatial exposure to the observation process. This could also arise from effects caused by shared tag properties affecting detectability and reliability of tags (e.g., batches of tags tags with shared manufacturing defects).	Include random effects for tagging cohort, capture site, or migratory route; model shared spatial processes explicitly; account for group-level correlation structure in detection and survival parameters.	[63, 73, 74]

#### Spatial heterogeneity in receiver arrays

**Fig. 2:**
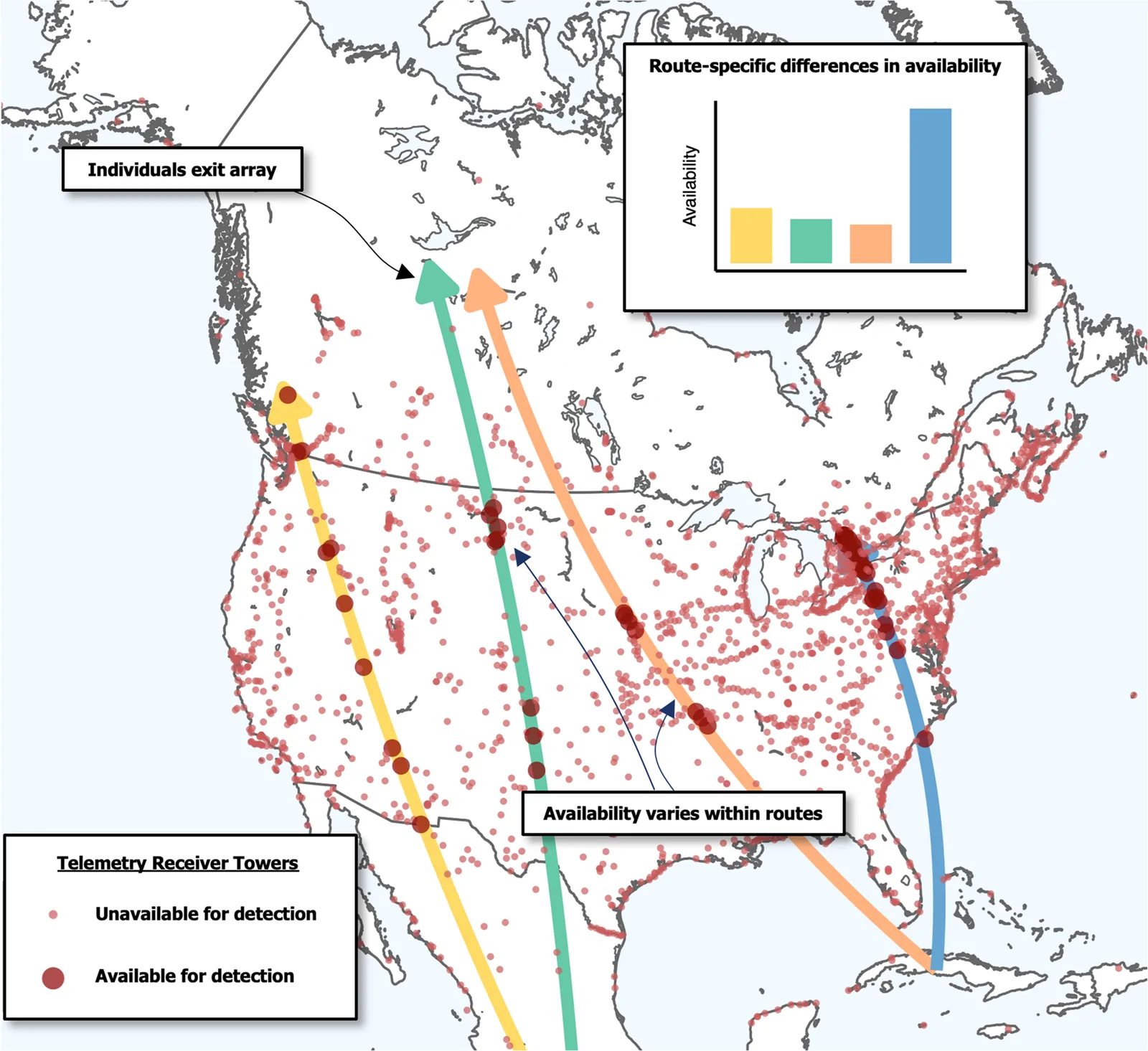
Hypothetical migratory routes through the Motus automated telemetry array illustrate violations of assumptions of the CJS model. Each arrow represents a hypothetical individual of a widespread species migrating northward where it is either available for detection (large, dark red points) or unavailable for detection (small, light red points). We considered a simulated individual to be “available” when its simulated track passed within 20 km of a receiver, an optimistic upper estimate for the detection range of Motus towers (Taylor et al. 2017). Each individual differs in the number of receivers at which it was available for potential detection (bar chart) and two individuals (green and orange) exit the array entirely. Finally, three individuals (green, orange, and blue) show significant clustering of receivers at which they were available, illustrating non-Markovian temporary emigration wherein having been available for detection at one time step results in a higher probability of being available at subsequent time (and vice versa)

Automated telemetry networks like OTN and Motus are primarily bottom-up, community-driven partnerships of individual researchers or research organizations, resulting in highly irregular networks of receivers (Fig. 1). Temporal coverage is similarly variable, as receivers are continually deployed and decommissioned when projects begin and end, or temporarily removed due to equipment failures or power issues. Existing telemetry networks are thus characterized by highly variable receiver coverage across both space and time. This heterogeneity means that individuals taking different routes through an array are differentially available for detection (Fig. 2), in violation of assumption 1. Heterogeneity in detection probability is known to bias estimates of apparent survival [63, 75, 76].

Even when individuals share common areas during parts of the annual cycle, they may exhibit different seasonal movement patterns through migratory connectivity [77]. For instance, individuals tagged in the same non-breeding areas may migrate to different breeding areas or vice versa (e.g. [78]), necessitating movement through areas with different densities of receivers (Fig. 2). In estuaries, individual fish may remain resident or migrate depending on physiological condition at the juvenile stage [79]. Even with shared breeding and non-breeding destinations, variation in migratory routes among individuals (e.g., [80]), can create substantial differences in detection opportunity (Fig. 2). For many species, network coverage varies by population, with some populations occurring partially or entirely outside the receiver array and thus having greater probability of permanent emigration from the detection network. For example, many North American migratory bird species have populations that breed north of existing Motus coverage in Canada and spend the non-breeding season south of coverage in South America (Fig. 1), and the destination of any individual in the population is often unknown. Similarly, dusky sharks (*Carcharhinus obscurus*) along the Atlantic coast of North America remain close to shore where acoustic receiver coverage is relatively high during their southward migration but the northward migration route appears to occur further offshore where few receivers exist [38]. Comparing survival across populations with different migratory destinations can therefore conflate true survival differences with differential emigration from the network, biasing apparent survival estimates low. Encountering this problem is even more likely when tracking young individuals, which often exhibit low natal philopatry (e.g., [81]),

Researchers comparing survival rates among groups or individuals must take care to ensure that availability is constant among groups, lest differences in processes like emigration lead to spurious inferences (see Supplementary Materials). Studies wherein inter-individual heterogeneity in availability is random with respect to the desired inference will not suffer from biased comparisons, because the rate of non-availability would be uniform across groups. By contrast, patterns of non-availability that are even partially confounded with the covariates being examined can lead to bias and spurious conclusions. For example, in migratory birds, environmental conditions on the breeding [82–87] and non-breeding grounds [81, 82] are both known to lead to large variation in dispersal distance and direction, and if dispersal varies among individuals or populations, then it becomes impossible to disentangle differences in survival from differences in availability.

#### Temporal dependence in observation process

It is also well established in the literature that estimates of apparent survival will be biased when availability at time *t* depends on detection status at *t*-1 [10, 48]. Because automated telemetry networks are often highly spatially clustered, detections occurring in dense portions of the array are more likely to be followed by detections on subsequent occasions and vice versa. This creates non-random patterns of availability over time that violate the assumption of independent detection probabilities which is fundamental to standard CMR models. Therefore, models must account for these patterns to avoid bias. Again, this problem is most severe in cases when inter-individual heterogeneity in movements exposes individuals to portions of the array with different degrees of receiver clustering. In these cases, the magnitude of the bias will vary among individuals exposed.

#### Continuous vs. discrete detection process

Traditional CMR data are generally collected during discrete, pre-defined sampling occasions which are often brief relative to the time between occasions. This sampling design allows researchers to define the time period over which apparent survival probabilities apply and ensures that variation in the time of captures within occasions is negligible relative to the time between occasions (assumption 4). In contrast, automated telemetry networks are characterized by a continuous detection process where the detection times themselves are random variables and the time between consecutive detections can vary widely within and among individuals. Although continuous-time CMR models exist [71], most automated telemetry studies aggregate detections into discrete occasions to use conventional discrete-time models (see Table 2). Imposing this discrete structure on the detections can lead to a mismatch between the data structure and the model assumptions, with important implications for survival estimation [88, 89]. For example, defining occasions as calendar days versus biologically meaningful periods (e.g., migration stages) can yield different survival estimates for the same dataset [70, 88]. When using discrete-time CMR models, researchers must decide how to aggregate continuous detection data into discrete occasions while acknowledging how different aggregation schemes can lead to different conclusions about survival. Alternatively, researchers may choose to use continuous-time formulations [71], though more work is needed to understand the strengths and weaknesses of discrete vs. continuous time survival models for automated telemetry data.

**Table 2:** Selected studies using CMR methods with automated telemetry with brief description of overall approach as well as approach to assumptions. Icons indicate:  = modified study design,   = modified analytical methods,  = no modifications

Study	Species	System	Region	Overall Approach	Approach to assumptions					
					#1	#2	#3	#4	#5	#6
Cooper et al. 2024 https://doi.org/10.1016/j.cub.2024.09.015 [17]	Kirtland’s Warbler *(Setophaga kirtlandii)*	Motus + manual	North America, Carribean	Used a novel, multistate CJS to estimate survival before, during, and after migration. Used “robust design” [48] to account for intra-individual non-Markovian availability. Relied on restricted ranges to control for inter-individual heterogeneity in migratory routes and destination.						
Blain et al. 2024 https://doi.org/10.1111/ele.14420 [90]	Swainson’s Thrush *(Catharus ustulatus)*	Motus	North America, South America	Used a standard CJS and a multi-state CJS to estimate survival across ancestry and heterozygosity in a hybrid contact zone. No controls for intra- or inter-individual heterogeneity in migratory routes.						
Blain et al. 2026 https://doi.org/10.1093/jeb/voaf148 [91]	Swainson’s Thrush *(Catharus ustulatus)*	Motus	North America, South America	Used a standard CJS and a multi-state CJS to estimate survival across ancestry and heterozygosity in a hybrid contact zone. No controls for intra- or inter-individual heterogeneity in migratory routes. (same system as above)						
Vastani et al. 2025 https://doi.org/10.21203/rs.3.rs-8108392/v1 [92]	Swainson’s Thrush *(Catharus ustulatus)*	Motus	North America, South America	Applied machine learning methods to survival estimation validated against CJS estimates from methods matching Blain et al. 2024.						
Evans et al. 2020 https://doi.org/10.1111/ibi.12727 [93]	Barn Swallow (*Hirundo rustica*)	Motus	North America	Used a multi-state “robust design” approach to estimate post-fledging survival at regional scale (60,000 km^2^). Also assessed effects of tag loss.						
Hayes et al. 2024 https://doi.org/10.1093/ornithapp/duad054 [54]	Wood Thrush (*Hylocichla mustelina*)	Motus + manual	North America	Used a combination of manual and automated radio-telemetry to estimate survival during post-fledging period, fall migration to breeding period, and the full year with the Burnham model [94] which mixes known- and unknown-fate approaches.						
Boyd et al. 2023 https://doi.org/10.1093/ornithapp/duad028 [95]	Wood Thrush (*Hylocichla mustelina*)	Motus + manual + aerial	North America	Used a combination of automated and manual telemetry surveys (terrestrial and aerial) to estimate survival from start of fall migration to breeding. No formal survival modeling accounting for probability of detection or heterogeneous availability, instead relying on naive detections as unbiased estimates of survival.						
Ward et al. 2018 https://doi.org/10.1098/rspb.2018.1747 [74]	Multiple	Custom	North America	Used a custom array to estimate survival of migrants crossing the Gulf of Mexico with a modified “robust design” approach [48].						
Hightower et al. 2015 https://doi.org/10.1080/19425120.2015.1088491 [60]	Atlantic sturgeon (*Acipenser oxyrinchus oxyrinchus*)	Atlantic Coastal Telemetry Network + Custom	North America	Used a custom array paired with a regional network to estimate survival in several major river systems in southeastern US.						
Aarestrup et al. 2014 https://doi.org/10.3354/meps10614 [59]	Sea Trout (*Salmo trutta*)	Custom + Manual	Europe	Used a custom array paired with manual tracking in a single river system to estimate survival without correction for probability of detection or movement.						
Flávio et al. 2020 https://doi.org/10.1111/fme.12405 [62]	Atlantic salmon (*Salmo salar*)	Custom	Europe	Used a custom array paired with manual tracking in a single river system to estimate survival without correction for probability of detection or movement.						
Melnychuck et al. 2018 https://doi.org/10.1093/beheco/ary119 [61]	Multiple salmon spp.	Ocean Tracking Network	North America	Used OTN compatible receivers to estimate migration survival in multiple river systems for 4 species of salmon without correcting for non-detection						
Gómez et al. 2026 https://doi.org/10.1093/ornithology/ukag050 [96]	Blackpoll Warbler (*Setophaga striata*)	Motus	North America, South America	Used a novel continuous-time multistate CJS to estimate survival before, during, and after migration. Used latitudinal bins to account for shared spatial variation in availability (but not among-individual heterogeneity within bins). No method for addressing for intra-individual non-Markovian availability. Relied on restricted range of tracked population to control for inter-individual heterogeneity in migratory routes.						

#### Additional sources of individual heterogeneity in detection and tag performance

Based on extensive collective experience working with automated telemetry data, we acknowledge that a large and often underappreciated source of bias arises from individual-level heterogeneity in detection probability conditional on availability. Thus, automated telemetry datasets also violate the assumption that individuals share a common detection probability. Unlike traditional capture mark recapture studies, where detection heterogeneity is often driven by behavioral responses to capture or observer effort, heterogeneity in automated telemetry arises from a combination of transmitter performance (e.g., pulse rate and strength), animal behavior, and individual morphology that is rarely observed directly.

A primary source of heterogeneity arises from persistent differences in detectability among individuals. Even among tags of identical model, antenna length, and nominal output power, realized transmission strength can vary due to manufacturing tolerances, antenna orientation, or attachment configuration [97, 98]. Because detection probability often declines steeply with effective signal strength, modest tag-level differences can translate into large differences in detection probability at a given distance, introducing persistent, unmeasured among-individual variation. Behavioral differences further compound this effect. In aerial systems, variation in flight altitude, route choice, or duration of flight bouts alter time spent within detectable range of receivers, while in aquatic systems, swimming depth, surfacing or burying behavior, and habitat use influence acoustic transmission and reception. These behaviors may be consistent among individuals or vary within individuals in response to environmental conditions, generating both among- and within-individual variation in detection probability across time. Finally, morphological and physiological traits such as body size, body composition, and tag orientation can interact with environmental and system-level factors to affect signal attenuation and reception for both radio and acoustic transmitters, producing systematic differences in detection across age classes, sexes, or populations [99, 100].

In addition to persistent heterogeneity, automated telemetry studies are affected by time-varying degradation in detectability associated with tag aging and failure [101]. Radio and acoustic transmitters have finite battery life, and tag performance near end-of-life can become unreliable, leading to reduced or intermittent detections that are observationally indistinguishable from mortality. This problem is particularly acute in studies that span a substantial fraction of expected tag life, where apparent survival estimates become increasingly difficult to interpret as tags approach their operational limits [102].

Discrete tag failure further exacerbates this problem. Physical tag loss can occur through attachment failure, mechanical damage, or predation events that separate the tag from the animal without killing it. Conversely, tags may remain intact after mortality events and continue to be detected, though such detections are often spatially restricted and less likely to generate spurious survival histories than tag failure or loss. Some tags are specifically designed to identify predation events, but variability in event detection, retention in the predator’s gut, and false positives may also lead to spurious survival histories [103]. In addition, tag performance can degrade abruptly, producing false absences that are observationally indistinguishable from death in encounter histories. When such failures occur randomly, apparent survival is biased low relative to true survival. When failure rates vary among individuals, populations, attachment methods, or environments, they introduce structured heterogeneity that can further confound inference about individual- or group-level differences in survival.

Together, persistent detectability differences, tag degradation, false positives in predation tags, and discrete tag failures generate strong individual-level heterogeneity in detection that can potentially be non-random with respect to encounter history. Individuals with lower or declining detectability are more likely to generate early terminal encounter histories and appear to die sooner, even when true survival does not differ. In sparse and spatially clustered telemetry networks with continuous detection, apparent survival may therefore reflect a mixture of mortality, movement, detectability, and tag performance rather than true demographic processes. Despite the central role of these mechanisms, their magnitude and relative importance remain poorly characterized in most automated telemetry systems, underscoring the need for explicit empirical and analytical work to quantify individual-level detection and tag-performance heterogeneity.

#### Simulation example

In the Supplementary Material we simulate a stylized example demonstrating the bias arising from the several of the processes described above. Briefly, we imagined a single, widely-distributed species whose pre-breeding migration traverses North America following four major flyways (as in Fig. 2). Critically, we simulated homogenous survival rates across all 4 flyways. Thus, the only difference in the populations is the portion of the receiver array that individuals traverse. We then estimated survival from these simulated detection histories (see Supplemental Material for detailed methods). We fit 3 survival models: 1) ϕ varies by flyway but *p* is held constant; 2) same as 1 but now *p* varies by flyway; and, 3) a naive model where flyways are lumped together along an East/West migratory divide and ϕ and *p* both vary by that divide. All 3 models produce substantial bias (both high and low) in estimates of ϕ relative to the known true survival rate. The models also inferred substantial differences in survival rates between flyways or regions which were entirely spurious. For example, simulation model 2, which allows by ϕ and *p* to vary by flyway, still failed to recover the true survival rate for 3 out of 4 flyways (on the basis of 95% CIs not overlapping the true survival rate), and incorrectly inferred several significant differences in survival rate between flyways. These simulations highlight the very real risk of spurious conclusions when failing to account for violations in the underlying assumptions of CJS models, even when attempting to account for these issues by allowing the probability of detection to vary along expected movement paths.

### Existing solutions and best practices

Despite these challenges, several methodological approaches show promise for estimating survival from automated telemetry data. We emphasize that the key is recognizing that for studies relying on irregular, ad hoc networks of automated telemetry receivers, standard CMR models are insufficient and specialized approaches are needed to account for the unique features of these data. Barring that, the use of standard “out of the box” CJS models will a priori violate multiple model assumptions and bias inference.

#### Spatially explicit approaches

There are methods available to correct for spatial availability bias by modeling a spatially explicit observation process. These approaches explicitly model the spatial configuration of the receiver array along with the movements of the marked animals to account for the heterogeneous probabilities that individuals will be available for detection among different locations. By incorporating information about receiver locations, detection ranges, and landscape features, these models can help separate true mortality from emigration outside the detection network [72, 104–106].

Implementation of these methods in the context of automated telemetry studies has the potential to alleviate many of the biggest hurdles described above because it would explicitly account for inter- and intra-individual variation in availability. Thus, these methods hold substantial promise to resolve the issues attendant to spatial and temporal heterogeneity in receiver networks. Recent work has begun to explicitly integrate telemetry data into spatial-capture-recapture approaches in order to more realistically model movement processes [65, 107, 108]. Implementing these methods in the context of automated telemetry systems would require a discrete-time movement model suitable for often directionally-persistent movements across a highly irregular automated telemetry network, which, to our knowledge, has not yet been developed. Developing such a model should be a high priority for researchers interested in using automated telemetry for survival estimation and would additionally benefit researchers interested in purely movement-based questions using these data.

#### Robust design

Non-random intra-individual heterogeneity in availability is a classic problem for CMR methods. This phenomenon can manifest in many ways, most commonly the “trap happy”/”trap shy” problem wherein an individual’s probability of capture following initial capture changes as the animal learns to seek or avoid the trap [48]. Introduced the so-called “robust design” CJS by subdividing capture occasions into series of “secondary occasions” which estimate two new parameters (typically *g*ʹ and *g*ʹʹ or γʹ and γʹʹ) which explicitly measure the probability of being unavailable at time *t* conditional on availability at time *t-1* [17]. Integrated the robust design into their multi-state CJS to account for this problem and found that *g*ʹ ≠ *g*ʹʹ, confirming that network irregularities can indeed produce this well described problem. Given that the robust design is so well understood and described and has been successfully used to estimate survival using automated telemetry data, it should be considered standard practice going forward. Non-random heterogeneity can also be accounted for in continuous-time formulations, for example, using Hawkes process models [71].

#### Multi-state models

Multi-state models extend the CJS framework by allowing individuals to occupy different discrete states (e.g., geographic regions, migratory stages), each with potentially different survival and detection probabilities. This structure can partition spatial heterogeneity in receiver coverage into observable states. For example, Melnychuk et al. (2017) [102] used multi-state models with acoustic telemetry to estimate seasonal survival in Atlantic sturgeon while accounting for spatially variable detection across receiver arrays, and Cooper et al. (2024) [17] used geographic states to estimate stage-specific survival in Kirtland’s Warblers while accounting for receiver density differences across regions (see below for further discussion). Multi-state models have also been used to account for acoustic telemetry tag failures in marine systems [69] but have not been widely applied in the terrestrial realm.

However, multi-state approaches (wherein the states serve as coarse approximations of individual location) are not an appropriate strategy to account for continuous movement through heterogeneous receiver coverage, which is the core challenge in most automated telemetry studies. Using multi-state survival models to capture animal movement processes through space would require forcing continuous spatial information into discrete states with sometimes arbitrary boundaries. Multi-state models also require relatively coarse state definitions in order to ensure sufficient detections within each state to estimate parameters. Finally, these models still confound permanent emigration with mortality. Despite these limitations, when combined with the robust design and applied to species with well-defined geographic or behavioral states, multi-state models can address some challenges of irregular networks.

#### Study design strategies

While careful study design can mitigate some challenges, opportunities to strategically design automated telemetry studies for survival estimation are limited. Unlike traditional CMR studies where researchers control trap placement and sampling effort, automated telemetry networks are largely community-driven, with receiver locations determined by independent research priorities, funding cycles, and logistical constraints. Some studies have successfully used “array augmentation” approaches, where manual tracking of range-restricted species is used to supplement automated detections in areas with sparse receiver coverage [17]. In marine environments, autonomous vehicles are increasingly used to fill gaps in receiver arrays [109, 110]. Coordinated regional or national-level efforts to expand telemetry networks offer some opportunities to integrate study design into future studies, though more work is needed to determine how best to augment existing networks to achieve specific study goals. For most current applications involving highly mobile species across continental-scale networks like Motus or OTN, study design alone cannot overcome fundamental issues of irregular coverage and heterogeneous availability. In these cases, appropriate analytical methods become essential rather than optional.

#### Data integration

Combining automated telemetry data with other data sources, in an integrated population modeling framework, could help constrain or bound parameters in the survival model because seasonal estimates are simultaneously informed by multiple data sources. For example, the violation of the aforementioned assumptions, in isolation, could lead to strong biases in survival estimates. Without additional data sources, those estimates may seem plausible. However, data integration techniques, such as Integrated Population Models (IPMs; a framework for linking disparate demographic datasets via a population balance equation [111, 112]), help constrain survival estimates during segments of the annual cycle into ecologically relevant parameter space [113]. In addition, data integration via an IPM-like framework would be flexible enough to embed other methodological elements as described above (e.g., robust design, multi-state models).

### Case studies: tractability of survival estimation across systems

To date, only a few studies have attempted to use automated telemetry to estimate survival during highly-mobile periods spanning a distributed receiver array (Table 2). Nevertheless, the literature provides both exemplary and cautionary examples of survival estimation from automated telemetry data. Here, we briefly review these studies to illustrate the importance of careful methodology and highlight the consequences of failing to account for observation processes.

#### Systems in which survival estimation is tractable

Hightower et al. [60] was the first study to estimate survival over a large spatial area using automated telemetry approaches applied to Atlantic sturgeon. This study developed methods that are still used in stock assessments for this wide-ranging and endangered species. In this case, survival estimation is possible because the sturgeon largely inhabit areas with high (albeit uneven) receiver coverage, are long-lived, and are large enough to carry tags with long battery life (frequently up to 10 years).

Ward et al. [74] was the first avian study to leverage automated telemetry approaches to estimate survival over large spatial scales. Specifically, they estimated survival rates for songbirds crossing the Gulf of Mexico during migration. Their success was partly due to the unique geography of their study system, where the Gulf crossing created a natural “funnel” that minimized availability bias. The study also benefited from intentionally high receiver density along the coast (a receiver “fence”), reducing the spatial heterogeneity that complicates survival estimation in other systems.

Cooper et al. [17] provide an example of how a combination of unique study design and model modifications can lead to successful survival estimation using Motus data. Specifically, this study estimated seasonal survival in Kirtland’s Warblers, a highly range-restricted species with negligible probability of permanent emigration from the receiver array [114, 115]. This study also implemented the robust design to account for spatial clustering in the receiver array and developed a novel multi-state framework that explicitly accounted for the directionally constrained movements attendant to migration. By carefully considering the observation process and using appropriate modeling techniques, they were able to demonstrate carry-over effects of non-breeding conditions on survival during migration.

In these cases, survival estimation was possible not because automated telemetry inherently resolves detectability challenges, but because key sources of heterogeneity in availability and detection were either minimized by study design, explicitly incorporated into the analytical framework, or both.

#### Systems in which survival estimation is fundamentally challenging

In contrast, estimating survival across the full annual cycle in widely distributed species presents substantially greater challenges. Species with broad breeding and non-breeding distributions, heterogeneous migratory routes, and uneven receiver coverage expose individuals to markedly different portions of the telemetry network. In such systems, inter-individual variation in availability and detection is intrinsically high and often strongly confounded with population identity, migratory strategy, or geographic origin.

Three recent studies have attempted to estimate survival across the annual cycle in a widely distributed species of bird, a much more challenging task than the geographically restricted examples described in the previous section. These three studies [90–92] estimated survival in the widely-distributed Swainson’s thrush (*Catharus swainsoni*) and exhibit several of the problems we have outlined in this paper, including: ignoring spatial and temporal patterns in receiver distribution while examining survival differences among populations of unknown breeding origin, that use different migratory routes (though note that these authors built a custom tower “fence” near the capture sites to mitigate some of these issues). Specifically, these studies examine differences in survival among populations (and hybrids) across a migratory divide. However, because population membership is confounded with migratory route and destination, inferences about differences in survival cannot be disentangled from possible differences in the availability process. In this case, the known density of towers varies substantially among the expected migratory routes (see [90] Fig. 1) and will lead to differences in availability among the populations. Individual thrushes may also exit the array entirely en route to final destinations, thus, becoming “permanently unavailable” which will bias ϕ low relative to true survival. The rate of permanent emigration via this process is also a priori expected to vary among populations based on low philopatry of the juvenile thrushes (~10–25%) [116, 117] and substantial heterogeneity in non-breeding sites used by hybrids that span nearly the entire longitudinal range of parentals [118]. To account for these issues, the authors let the probability of detection vary by genetic proxies for population (and, thus, putative migratory route and destination). However, this cannot fully address the bias introduced by permanent emigration and the highly irregular array (see *Simulation Example* above and Supplementary Material). Thus, the differences in survival estimates presented in these papers cannot be uniquely attributed to survival per se because the known heterogeneity in migratory routes is expected to produce similar results due to differential availability alone.

These examples underscore a key limitation of current approaches; When spatial heterogeneity in receiver coverage aligns with biological structure, such as population-specific migratory pathways, standard capture–mark–recapture models lack sufficient information to separate survival from detection and movement processes. In such cases, strong inference about survival is not possible without additional constraints, auxiliary data, or methodological advances.

### Future directions and recommendations

#### Methodological development priorities

Efforts to estimate survival using automated telemetry networks would benefit from continued development of analytical methods specifically designed for the unique attributes of these data. Integration of movement and survival modeling represents an important frontier [3, 4, 119, 120]. Because survival and movement are intimately linked processes, joint modeling approaches that consider both simultaneously may provide more accurate and biologically meaningful estimates than models that treat them separately. Similarly, data integration with other related demographic datasets (sensu [121]) could help to constrain estimates derived from automated telemetry data to biologically realistic ranges.

Additional methodological priorities include the development of simulation tools to support study design and inference. Simulation frameworks that allow users to specify receiver network geometry, tag properties, and realistic movement behaviors would enable researchers to evaluate whether survival estimation is feasible for a given system and to quantify expected bias under alternative designs. Closely related is the need for benchmark datasets and evaluation protocols that allow new analytical methods to be tested and compared under standardized conditions. Finally, the field would benefit from the establishment of clear diagnostic criteria for assessing whether a given automated telemetry dataset contains sufficient information to support survival estimation, analogous to goodness-of-fit and identifiability checks used in traditional capture–mark–recapture analyses.

#### Study design guidelines

Researchers planning to use automated telemetry data for survival estimation should carefully consider several design aspects before beginning data collection. Array coverage should be evaluated relative to the movement patterns of target species, with particular attention to ensuring adequate (and relatively similar) coverage of key habitats and movement corridors to maintain availability across individuals included in a study. Power analyses should be conducted to determine appropriate sample sizes, and consideration should be given to integrating automated telemetry with other monitoring methods.

We recommend that researchers conduct preliminary analyses to assess the adequacy of receiver coverage for their specific questions. This might include simulation studies that evaluate the expected bias in survival estimates given the spatial configuration of available receivers and the movement behavior of target species [122].

#### Network development

Continued expansion and strategic enhancement of automated telemetry networks will be crucial for advancing survival estimation capabilities. Priority areas for network development include filling coverage gaps in key migration corridors, improving international coordination for migratory species, and integrating automated telemetry with complementary technologies such as GPS tracking and environmental monitoring. To some extent, ad hoc networks of researchers have begun this work by deploying receiving stations in arrangements designed to resemble fences (e.g., Northeast Motus Network).

Technology improvements that extend tag life will also enhance the utility of automated telemetry for survival studies. Relatedly, the detection range of tags is only poorly understood and more studies examining how detection varies not only by distance to tower but with other factors such as topography, animal behavior, weather conditions, or even morphology are sorely needed. Finally, real-time data processing capabilities could enable adaptive study designs that respond to patterns observed during data collection.

## Conclusions

Automated telemetry networks like OTN and Motus represent a revolutionary advance in our ability to track the movements and fates of small, highly-mobile organisms. However, realizing the potential of these systems for survival estimation requires careful attention to the observation processes that generate detection data. The irregular spatial distribution of receivers, continuous nature of detection processes, and the complexity of tag performance all create challenges that are not adequately addressed by standard methods for survival estimation (i.e., CMR models).

Successfully estimating survival from automated telemetry requires recognizing when standard CJS models are inadequate, implementing modifications like the robust design or spatially explicit frameworks, and acknowledging the limitations imposed by irregular receiver coverage. The examples of successful applications demonstrate that these challenges are not insurmountable, but they do require specialized approaches and careful consideration of system limitations.

As automated telemetry networks continue to expand and technology continues to improve, the opportunities for advancing our understanding of survival processes will only grow. However, methodological development must keep pace with technological advances. Investment in method development, training, and capacity building will be essential to ensure that the full potential of these remarkable systems is realized.

For most highly-mobile species, estimating survival will likely require increased spatial coverage of receiver stations paired with methodological advances. In the interim, researchers must critically evaluate whether apparent differences in survival reflect true mortality patterns or simply heterogeneity in detection opportunity. The stakes are high: survival estimation is fundamental to both basic ecological understanding and practical conservation applications. Getting it right matters not just for the advancement of basic research, but for the effective conservation of the species these systems are designed to help us understand and protect.

## Electronic supplementary material

Below is the link to the electronic supplementary material.

Supplementary Material 1

## Data Availability

No datasets were generated or analysed during the current study.
