# Supplementary material for: From detections to demography: methodological challenges and opportunities in survival estimation from automated telemetry networks
Source: Mov Ecol. 2026 Nov 5;14:79. doi: 10.1186/s40462-026-00711-4 (PMC13737629; doi:10.1186/s40462-026-00711-4)
Supplement: Supplementary file 1 [file 40462_2026_711_MOESM1_ESM.pdf]

Supplementary Material

Motus Survival Simulation

```r
# Set seed for reproducibility
set.seed(4444)
# Load packages
library(motus)
library(tidyverse)
```

```
── Attaching core tidyverse packages ──────────────────────── tidyverse 2.0.0 ──
✔ dplyr     1.2.1     ✔ readr     2.2.0
✔ forcats   1.0.1     ✔ stringr   1.6.0
✔ ggplot2   4.0.3     ✔ tibble    3.3.1
✔ lubridate 1.9.5     ✔ tidyr     1.3.2
✔ purrr     1.2.2
── Conflicts ────────────────────────────────────────── tidyverse_conflicts()
──
✖ dplyr::filter() masks stats::filter()
✖ dplyr::lag()    masks stats::lag()
ℹ Use the conflicted package (<http://conflicted.r-lib.org/>) to force all
conflicts to become errors
```

```r
library(ggplot2)
library(sf)
```

```
Linking to GEOS 3.13.0, GDAL 3.8.5, PROJ 9.5.1; sf_use_s2() is TRUE
```

```r
library(rnaturalearth)
library(rnaturalearthdata)
```

```


Attaching package: 'rnaturalearthdata'

The following object is masked from 'package:rnaturalearth':

    countries110
```

```
library(lubridate)
library(marked)
```

```
Loading required package: lme4
Loading required package: Matrix

Attaching package: 'Matrix'

The following objects are masked from 'package:tidyr':

    expand, pack, unpack

Loading required package: parallel
This is marked 1.2.8
```

```
library(glue)
```

Here, using a simple simulation, we show how spatial variance in movements can lead to biased survival estimates in simle Cormack-Jolly-Seber (CJS) models. To do this we imagine a winde-spread migratory bird moving northbound within four “flyways” during migration and crossing the Motus array.

Simulate Data

First we define a fucntion to simulate movement routes. Routes are conceived of as continous curved paths whoese endpoints and curvature can be user-specified. Each route is initially described using 100 points.

```
# Function to create a curved path with optional random variation
create_curved_path <- function(
    x_start, y_start,
    x_end, y_end,
    curvature,
    n_points = 100,
    endpoint_sd = 0,
    curvature_sd = 0
) {

  # Randomly vary start and end points
  x_start <- x_start + rnorm(1, mean = 0, sd = endpoint_sd)
  y_start <- y_start + rnorm(1, mean = 0, sd = endpoint_sd)
```

```
  x_end <- x_end + rnorm(1, mean = 0, sd = endpoint_sd)
  y_end <- y_end + rnorm(1, mean = 0, sd = endpoint_sd)

  # Randomly vary curvature
  curvature <- curvature + rnorm(1, mean = 0, sd = curvature_sd)

  # Generate points along the curve
  t <- seq(0, 1, length.out = n_points)

  # Calculate control point for quadratic "Bezier" curve
  mx <- (x_start + x_end) / 2
  my <- (y_start + y_end) / 2

  dx <- x_end - x_start
  dy <- y_end - y_start
  dist <- sqrt(dx^2 + dy^2)

  cx <- mx - curvature * dist * dy / dist
  cy <- my + curvature * dist * dx / dist

  # Quadratic Bezier formula
  x <- (1 - t)^2 * x_start +
    2 * (1 - t) * t * cx +
    t^2 * x_end

  y <- (1 - t)^2 * y_start +
    2 * (1 - t) * t * cy +
    t^2 * y_end

  # Return as linestring
  st_linestring(cbind(x, y))
}
```

We now simulate tracks within four typified "flyways" - tracks vary randomly around the typified flyway route.

```
# Create arrow coordinates for flyways in lat/long then transform
arrow_coords <- data.frame(
  route = c("central", "central", "east", "east", "mexico", "mexico", "pacific",
"pacific"),
  point = c("start", "end", "start", "end", "start", "end", "start", "end"),
  lon = c(-80, -110, -80, -80, -100, -115, -102, -125),
  lat = c(22, 65, 22, 45, 18, 65, 18, 52)
```

```
) %>%
  st_as_sf(coords = c("lon", "lat"), crs = 4326) %>%
  st_transform(crs = 2163) %>%
  mutate(x = st_coordinates(.)[,1],
         y = st_coordinates(.)[,2]) %>%
  st_drop_geometry()
```

```
Warning in CPL_crs_from_input(x): GDAL Message 1: CRS EPSG:2163 is deprecated.
Its non-deprecated replacement EPSG:9311 will be used instead. To use the
original CRS, set the OSR_USE_NON_DEPRECATED configuration option to NO.
```

```
# Reshape for geom_curve
arrows <- arrow_coords %>%
  pivot_wider(names_from = point, values_from = c(x, y))

# declare flyway specific curvature
flyway_curvature <- c(0.15, -0.15, -0.05, 0.25) # flyway specific curvature

# declare n ind per "flyway"
n_individuals <- 500

# Simulate individual paths
paths <- data.frame(
  route = c("central", "east", "mexico", "pacific"),
  curvature = flyway_curvature
) %>%
  left_join(arrows, by = "route") %>%
  mutate(n = n_individuals) %>%
  tidyr::uncount(n, .id = "individual") %>%
  mutate(
    individual_id = paste(route, individual, sep = "_")
  ) %>%
  rowwise() %>%
  mutate(
    geometry = list(
      create_curved_path(
        x_start,
        y_start,
        x_end,
        y_end,
        curvature,
        endpoint_sd = 50000,  # 10 km variation in endpoints
```

```
        curvature_sd = 0.03   # small variation in route curvature
      )
    )
  ) %>%
  ungroup() %>%
  st_sf(crs = 2163)
```

Next we convert the linestring representations of each individual track into discrete time. This is something like the expected location for that route at each sample occasion (disregarding survival and observation processes still).

```
# Number of steps per path
n_occasions <- 25

track_points <- paths %>%
  mutate(
    point_data = map(
      geometry,
      function(g) {
        xy <- st_coordinates(g)

        # Select evenly spaced points along the full track
        occasion_index <- round(
          seq(1, nrow(xy), length.out = n_occasions)
        )
        #
        tibble(
          occasion = seq_len(n_occasions),
          x = xy[occasion_index, 1],
          y = xy[occasion_index, 2]
        )
      }
    )
  ) %>%
  st_drop_geometry() %>%
  select(individual_id, route, point_data) %>%
  unnest(point_data) %>%
  mutate(
    population = route
  ) %>%
  st_as_sf(
    coords = c("x", "y"),
```

```
    crs = 2163
  )
```

At this stage we can simulate the survival process whereby we generate an alive/dead status at each occasion. Critically, for the purposes of this simulation we assume *homogeneous survival* across all individuals. In other words, for the remainder of this simulation, every individual and flyway has the same true probability of survival (ϕ)

```
# Overall probability of surviving the complete migration
phi_route <- 0.4
#
# Constant survival probability per discrete interval
print("True probability of surviving from occasion t to t+1:")
```

```
[1] "True probability of surviving from occasion t to t+1:"
```

```
(phi_step <- phi_route^(1 / (n_occasions - 1)))
```

```
[1] 0.9625408
```

```
# Simulate survival process
track_points <- track_points %>%
  arrange(individual_id, occasion) %>%
  group_by(individual_id) %>%
  mutate(
    survived_interval = c(
      1L,
      rbinom(
        n() - 1,
        size = 1,
        prob = phi_step
      )
    ),
     #convert independent "transition" probabilities to a permanent alive dead
state
    alive = cumprod(survived_interval)
  ) %>%
  ungroup()
```

On top of the simulated survival process, we can now simulate the automated telemetry detection process. For simplicity, we conservatively assume that any individual in an alive state that is within 20 km of a tower is detected.

```
# Load Motus tower dats data
tbl_recvDeps <- read_csv("../out/recv-deps.csv")
```

```
New names:
Rows: 8768 Columns: 22
── Column specification
──────────────────────────────────────────────────────── Delimiter: "," chr
(7): serno, receiverType, status, name, siteName, stationName, fixture... dbl
(12): ...1, deployID, projectID, deviceID, latitude, longitude, elevati... lgl
(1): macAddress dttm (2): start_date, end_date
ℹ Use `spec()` to retrieve the full column specification for this data. ℹ
Specify the column types or set `show_col_types = FALSE` to quiet this message.
• `` -> `...1`
```

```
# Remove stations with missing spatial information
stations_clean <- tbl_recvDeps %>%
  filter(!is.na(latitude) & !is.na(longitude)) %>%
  filter(latitude != 0 & longitude != 0)

# Extract only active stations
stations_active <- stations_clean %>%
  filter(status == "active")

# Convert stations to sf object and transform
stations_active_sf <- st_as_sf(stations_active,
                               coords = c("longitude", "latitude"),
                               crs = 4326) %>%
  st_transform(crs = 2163)

# Tower detection radius
detection_radius <- 20000

# Check distance from tower for all locations
near_tower <- lengths(
  st_is_within_distance(
    track_points,
    stations_active_sf,
    dist = detection_radius
```

```
  )
) > 0

# Add detection status to track_points
track_points <- track_points %>%
  mutate(
    near_tower = near_tower, #add near tower state
    detected = as.integer( # detected if...
      alive == 1 & near_tower # alive, and near tower
    ),

    # Initial marking occasion is known with certainty
    detected = if_else(
      occasion == 1,
      1L,
      detected
    )
  )
```

We can now plot the simulated tracks, showing only the detections of live individuals.

```
# Load some background data for plotting
# Get world map
world <- ne_countries(scale = "large", returnclass = "sf")
world_proj <- st_transform(world, crs = 2163)

# Create bounding box for ocean background
ocean <- st_as_sfc(st_bbox(c(xmin = -4500000, xmax = 7000000,
                             ymin = -7000000, ymax = 4000000),
                           crs = st_crs(2163)))

lakes <- ne_download(scale = "medium", type = "lakes", category = "physical",
returnclass = "sf")
```

```
Reading 'ne_50m_lakes.zip' from naturalearth...
```

```
lakes_proj <- st_transform(lakes, crs = 2163)

# Keep only observed Motus detections
detected_points <- track_points %>%
  filter(
    detected == 1
```

```r
  ) %>%
  arrange(individual_id, occasion)

# Convert to lines
detected_lines <- detected_points %>%
  group_by(individual_id, route) %>%
  filter(n() >= 2) %>%
  summarise(
    geometry = st_combine(geometry),
    .groups = "drop"
  ) %>%
  st_cast("LINESTRING")

observed_track_map <- ggplot() +
  geom_sf(
    data = ocean,
    fill = "aliceblue",
    color = NA
  ) +
  geom_sf(
    data = world_proj,
    fill = "white",
    color = "grey40",
    linewidth = 0.4
  ) +
  geom_sf(
    data = lakes_proj,
    fill = "aliceblue",
    color = "grey40",
    linewidth = 0.3
  ) +

  # Lines reconstructed from successive detections only
  geom_sf(
    data = detected_lines,
    aes(color = route),
    linewidth = 0.5,
    alpha = 0.5
  ) +

  # Actual detected locations
  geom_sf(
    data = detected_points,
```

```
    aes(color = route),
    size = 1.2,
    alpha = 0.8
  ) +

  # Motus stations
  geom_sf(
    data = stations_active_sf,
    color = "black",
    size = 0.3,
    alpha = 0.35
  ) +
  scale_color_manual(
    values = c(
      west = "#ffb380",
      east = "#66a3d2",
      mexico = "#66c9a8",
      pacific = "#ffd966"
    ),
    name = "Route"
  ) +
  coord_sf(
    xlim = c(-3000000, 3000000),
    ylim = c(-3500000, 3000000)
  ) +
  theme_void() +
  theme(
    legend.position = "bottom"
  )

observed_track_map
```

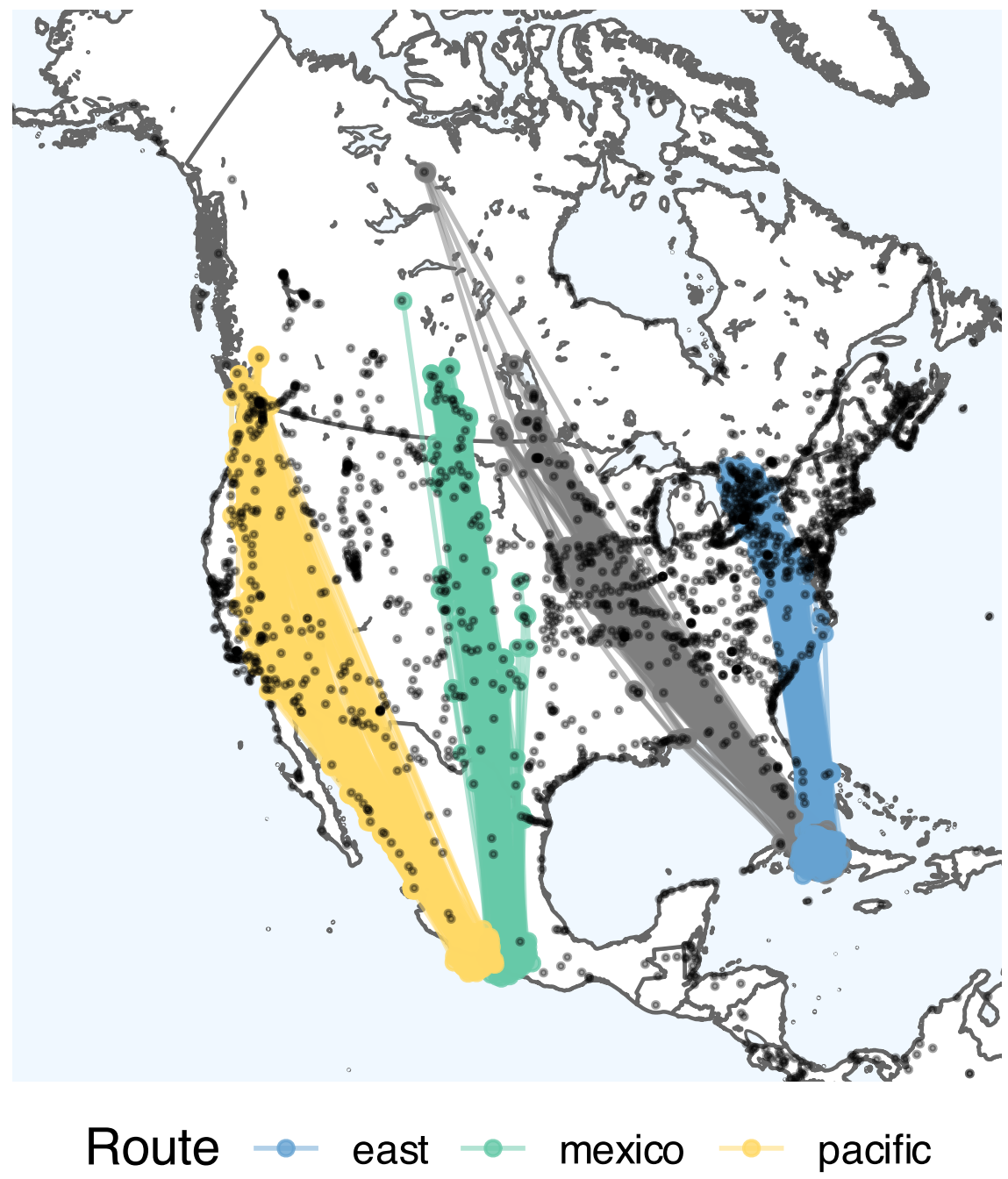

Survival Models

Below we fit 3 survival models:
1. A naive model that estimates flyway-specific survival, but assumes constant probability of detection (p) across flyways;
2. A model that relaxes the assumption of homogeneous p by allowing probability of detection to vary by flyway; and,
3. A model that only includes 2 groupings based on the simulated east-west migratory divide (which also corresponds to the simulated banding locations)

Recall that in all cases true survival is the same among the flyways so an unbiased estimate should retun no differences among groups.

First we arrange the data as a capture

```
cjs_data <- track_points %>%
  st_drop_geometry() %>%
  mutate(population = case_when(route == "east" ~ "east",
                               route == "central" ~ "east",
                               route == "mexico" ~ "west",
                               route == "pacific" ~ "west")) %>%

  group_by(
    individual_id,
    population,
    route
```

```r
  ) %>%
  summarise(
    ch = paste0(detected, collapse = ""),
    freq = 1L,
    .groups = "drop"
  )

head(cjs_data)
```

```
# A tibble: 6 × 5
  individual_id population route   ch                        freq
  <chr>         <chr>      <chr>   <chr>                    <int>
1 central_1     east       central 1000000101000000000000000     1
2 central_10    east       central 1000000000010000000000000     1
3 central_100   east       central 1000000000000000000000000     1
4 central_101   east       central 1000000001100000000000000     1
5 central_102   east       central 1000000000000000000000000     1
6 central_103   east       central 1000000000000000000000000     1
```

Model 1: ϕ ~ flyway, p ~ 1

Here we fit a model that estimates flyway-specific survival probability but naively treats the probability of detection as homogeneous across individuals.

```r
# prep the data into a format suitable for `marked`:
cjs_mark_data <- cjs_data %>%
  select(ch, freq, route) %>%
  mutate(route = factor(route)) %>%
  transmute(
    ch = as.character(ch),
    freq = as.numeric(freq),
    route = factor(route)
  ) %>%
  as.data.frame()

# Fit the model
cjs_model_1 <- crm(
  data = cjs_mark_data,
  model = "CJS",
  model.parameters = list(
    Phi = list(formula = ~ 0 + route),
    p   = list(formula = ~ 1)
  ),
```

```
  hessian = TRUE
)
```

```
2000 capture histories collapsed into 499
```

```
Warning: the 'nobars' function has moved to the reformulas package. Please update
your imports, or ask an upstream package maintainer to do so.
This warning is displayed once per session.
```

```
Warning: the 'findbars' function has moved to the reformulas package. Please
update your imports, or ask an upstream package maintainer to do so.
This warning is displayed once per session.
```

```
Computing initial parameter estimates
```

```
Accumulating capture histories based on design. This can take awhile...
```

```
499 capture histories collapsed into 499
```

```
Starting optimization for 5 parameters...
```

```


 Number of evaluations:  100  -2lnl: 20275.23626
 Number of evaluations:  200  -2lnl: 20282.54386
 Number of evaluations:  300  -2lnl: 20275.08037
```

```
Computing hessian...
```

```


 Number of evaluations:  100  -2lnl: 20410.83728
```

```


Elapsed time in minutes:  0.0102
```

Now we convert the parameter estimates to the probability scale and assess whether the known-true survival is contined within the bounds of the estimated confidence intervals.

```
route_predictions_1 <- predict(
  cjs_model_1,
  newdata = data.frame(
    route = factor(
      levels(cjs_mark_data$route),
      levels = levels(cjs_mark_data$route)
    )
  ),
  se = TRUE
)

print(glue("True survival = {phi_step}"))
```

```
True survival = 0.962540841979741
```

```
route_predictions_1$Phi |>
  mutate(error_flag = ifelse(lcl <= phi_step & ucl >= phi_step, 0, 1))
```

```
    route occ  estimate          se       lcl       ucl error_flag
1 central   1 0.9381264 0.004370457 0.9289859 0.9461586          1
2    east   1 0.9816535 0.002233218 0.9767235 0.9855547          1
3  mexico   1 0.9438087 0.004107389 0.9351944 0.9513375          1
4 pacific   1 0.9786579 0.002513323 0.9731338 0.9830660          1
```

We see that all of the estimates were significantly different biased, differing from the known true survival probability used to generate the simulated data. Several of the estimates also differed from one another - notably the "Mexico" and "Central" flyways' survival estimates were substantially loweer tham those fro "East" and "Pacific". This arises primarily because the receiver array is far denser along the coasts resulting in greater availability, which is confounded with survival (i.e., apparent survival). While these differences may seem small, recall that this is the estimated survival over a single occasion interval. When these estimated probabilities are exponentiated to reveal estimated survival over the entire migration, the differences are much more substantial:

```
(p1 <- tibble(route = c("east", "mexico", "pacific", "central"),
              estimate = route_predictions_1$Phi$estimate^n_occasions,
              lcl = route_predictions_1$Phi$lcl^n_occasions,
              ucl = route_predictions_1$Phi$ucl^n_occasions)
)
```

```
# A tibble: 4 × 4
  route   estimate   lcl   ucl
  <chr>      <dbl> <dbl> <dbl>
1 east       0.203 0.159 0.251
2 mexico     0.629 0.555 0.695
3 pacific    0.236 0.187 0.287
4 central    0.583 0.506 0.652
```

```
ggplot(data = p1, aes(x = route, y = estimate, ymin = lcl, ymax = ucl, color
= route))+
  geom_errorbar(width = 0.25, size = 2)+
  scale_color_manual(
    values = c(
      west = "#ffb380",
      east = "#66a3d2",
      mexico = "#66c9a8",
      pacific = "#ffd966"
    ),
    name = "Route"
  ) +
  geom_hline(aes(yintercept = phi_route), linetype = "dashed")+
  annotate(
    "text",
    x = Inf,
    y = phi_route,
    label = "True survival probability = 0.4",
    hjust = 1.05,
    vjust = -0.6,
    color = "black",
    size = 4
  ) +
  theme_classic()+
  theme(legend.position = "none")
```

```
Warning: Using `size` aesthetic for lines was deprecated in ggplot2 3.4.0.
ℹ Please use `linewidth` instead.
```

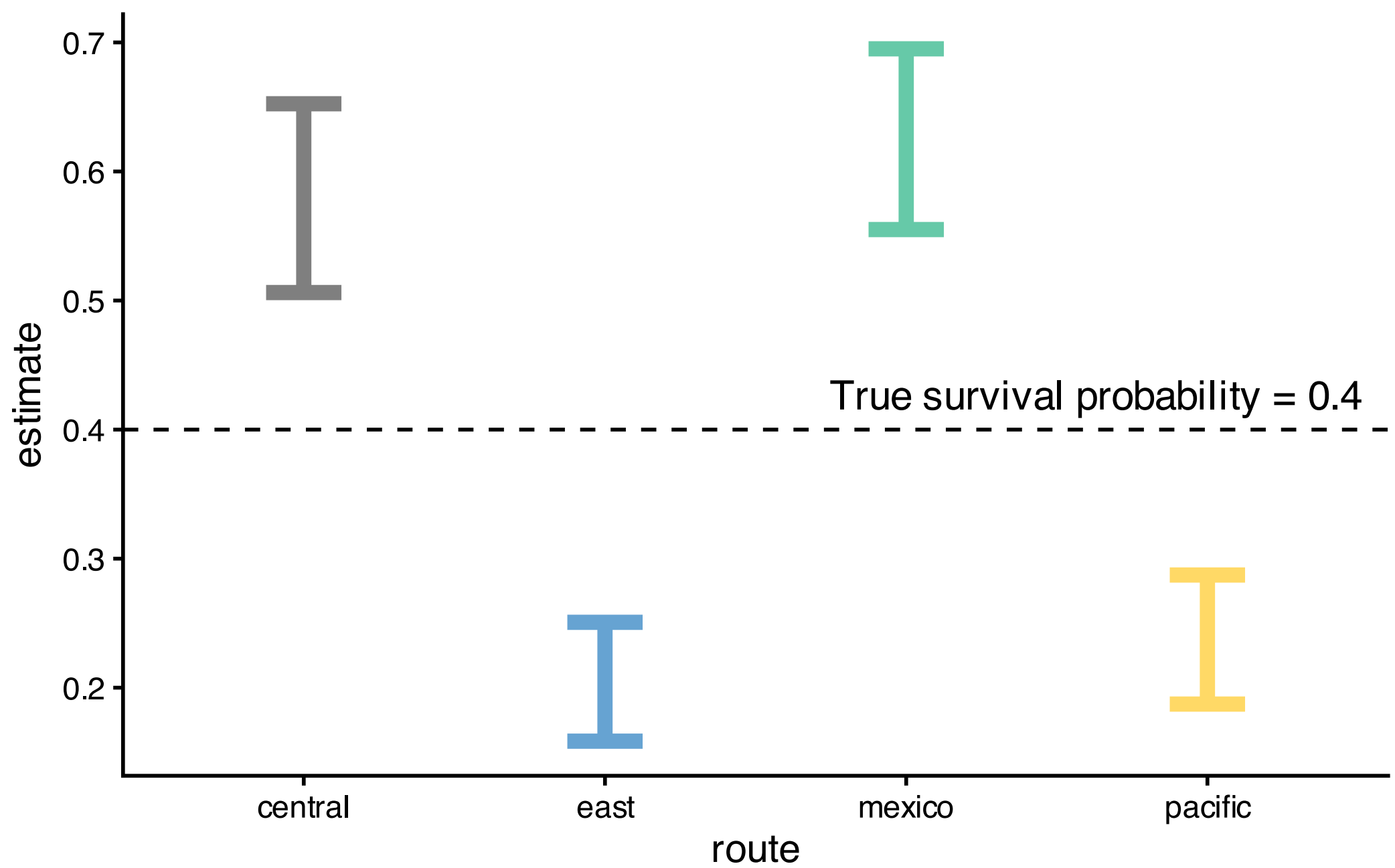

Model 2: ϕ ~ flyway, p ~ flyway

We now repeat that simulation but allow probability of detection (p) to vary by flyway. The logic here would be that incorporating this route-specific variance in detection will capture the spatial heterogeneity in availability.

```
# Fit the model
cjs_model_2 <- crm(
  data = cjs_mark_data,
  model = "CJS",
  model.parameters = list(
    Phi = list(formula = ~ 0 + route),
    p   = list(formula = ~ route)
  ),
  hessian = TRUE
)
```

```
2000 capture histories collapsed into 499
```

```
Computing initial parameter estimates
```

```
Accumulating capture histories based on design. This can take awhile...
```

```
499 capture histories collapsed into 499
```

```
Starting optimization for 8 parameters...
```

```
 Number of evaluations:  100  -2lnl: 20314.69727
 Number of evaluations:  200  -2lnl: 20068.49754
 Number of evaluations:  300  -2lnl: 20010.33017
 Number of evaluations:  400  -2lnl: 20010.34003
 Number of evaluations:  500  -2lnl: 20017.81548
 Number of evaluations:  600  -2lnl:  20010.4238
```

```
Computing hessian...
```

```
 Number of evaluations:  100  -2lnl: 20044.73111
 Number of evaluations:  200  -2lnl:  20011.7165
```

```
Elapsed time in minutes:  0.0125
```

Again we can see biased estimates of ϕ arise. While allowing p to vary by route does capture some of he variance in availability (thus, reducing some of the estimation bias) it still cannot full account for differences in permenent emigration and non-Markovian temporary emigration processes.

```
route_predictions_2 <- predict(
  cjs_model_2,
  newdata = data.frame(
    route = factor(
      levels(cjs_mark_data$route),
      levels = levels(cjs_mark_data$route)
    )
  ),
  se = TRUE
)

print(glue("True survival = {phi_step}"))
```

```
True survival = 0.962540841979741
```

```r
route_predictions_2$Phi |>
  mutate(error_flag = ifelse(lcl <= phi_step & ucl >= phi_step, 0, 1))
```

```
    route occ estimate          se       lcl       ucl error_flag
1 central   1 0.946495 0.005083617 0.9356100 0.9556272          1
2    east   1 0.970848 0.002288693 0.9660113 0.9750142          1
3  mexico   1 0.975509 0.005177378 0.9630275 0.9838475          1
4 pacific   1 0.982884 0.002890277 0.9761944 0.9877174          1
```

```r
(p2 <- tibble(route = c("east", "mexico", "pacific", "central"),
              estimate = route_predictions_2$Phi$estimate^n_occasions,
              lcl = route_predictions_2$Phi$lcl^n_occasions,
              ucl = route_predictions_2$Phi$ucl^n_occasions)
)
```

```
# A tibble: 4 × 4
  route   estimate   lcl   ucl
  <chr>      <dbl> <dbl> <dbl>
1 east       0.253 0.189 0.322
2 mexico     0.477 0.421 0.531
3 pacific    0.538 0.390 0.666
4 central    0.649 0.548 0.734
```

```r
ggplot(data = p2, aes(x = route, y = estimate, ymin = lcl, ymax = ucl, color
= route))+
  geom_errorbar(width = 0.25, size = 2)+
  scale_color_manual(
    values = c(
      west = "#ffb380",
      east = "#66a3d2",
      mexico = "#66c9a8",
      pacific = "#ffd966"
    ),
    name = "Route"
  ) +
  geom_hline(aes(yintercept = phi_route), linetype = "dashed")+
  annotate(
    "text",
    x = Inf,
    y = phi_route,
    label = "True survival probability = 0.4",
```

```
    hjust = 1.05,
    vjust = -0.6,
    color = "black",
    size = 4
  ) +
  theme_classic()+
  theme(legend.position = "none")
```

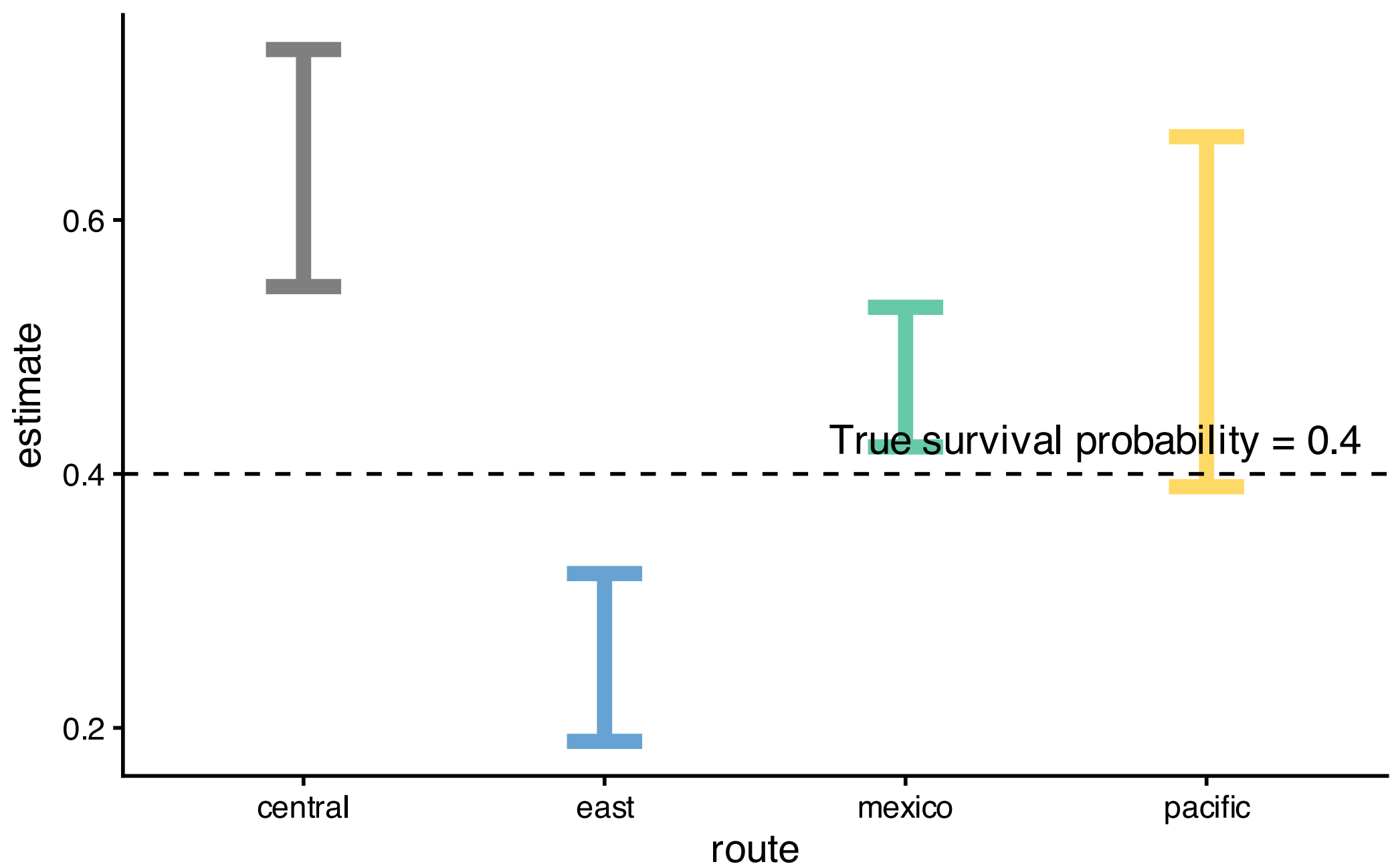

Model 3: ϕ ~ east/west, p ~ east/west

This model, while conceptually similar to Model 2, is perhaps more realistic in that it simulates a situation where researchers affix tags at two capture locations corresponding to a known migratory divide but do not allow parameters to vary more precisely with migraotry route (perhaps because it is only coarsly known a priori). Again, allowing the probability of detection to vary by population is insufficient to avoid biased estimates of ϕ for both populations.

```
# prep the data into a format suitable for `marked`:
cjs_mark_data <- cjs_data %>%
  select(ch, freq, population) %>%
  mutate(population = factor(population)) %>%
  transmute(
    ch = as.character(ch),
    freq = as.numeric(freq),
    population = factor(population)
```

```
  ) %>%
  as.data.frame()

# Fit the model
cjs_model_3 <- crm(
  data = cjs_mark_data,
  model = "CJS",
  model.parameters = list(
    Phi = list(formula = ~ 0 + population),
    p   = list(formula = ~ population)
  ),
  hessian = TRUE
)
```

```
2000 capture histories collapsed into 469
```

```
Computing initial parameter estimates
```

```
Accumulating capture histories based on design. This can take awhile...
```

```
469 capture histories collapsed into 469
```

```
Starting optimization for 4 parameters...
```

```


 Number of evaluations:  100  -2lnl: 20311.66679
 Number of evaluations:  200  -2lnl: 20311.80353
```

```
Computing hessian...
```

```


Elapsed time in minutes:  0.0087
```

```
route_predictions_3 <- predict(
  cjs_model_3,
  newdata = data.frame(
    population = factor(
      levels(cjs_mark_data$population),
```

```
      levels = levels(cjs_mark_data$population)
    )
  ),
  se = TRUE
)

print(glue("True survival = {phi_step}"))
```

```
True survival = 0.962540841979741
```

```
route_predictions_3$Phi |>
  mutate(error_flag = ifelse(lcl <= phi_step & ucl >= phi_step, 0, 1))
```

```
  population occ  estimate          se       lcl       ucl error_flag
1       east   1 0.9584786 0.002346102 0.9536287 0.9628410          0
2       west   1 0.9777296 0.002675093 0.9718370 0.9824116          1
```

```
(p3 <- tibble(population= c("east", "west"),
              estimate = route_predictions_3$Phi$estimate^n_occasions,
              lcl = route_predictions_3$Phi$lcl^n_occasions,
              ucl = route_predictions_3$Phi$ucl^n_occasions)
)
```

```
# A tibble: 2 × 4
  population estimate   lcl   ucl
  <chr>         <dbl> <dbl> <dbl>
1 east          0.346 0.305 0.388
2 west          0.569 0.490 0.642
```

```
ggplot(data = p3, aes(x = population, y = estimate, ymin = lcl, ymax = ucl, color
= population))+
  geom_errorbar(width = 0.25, size = 2)+
  scale_color_manual(
    values = c(
      west = "#ffb380",
      east = "#66a3d2"
    ),
    name = "Route"
  ) +
  geom_hline(aes(yintercept = phi_route), linetype = "dashed")+
```

```
  annotate(
    "text",
    x = Inf,
    y = phi_route,
    label = "True survival probability = 0.4",
    hjust = 1.05,
    vjust = -0.6,
    color = "black",
    size = 4
  ) +
  theme_classic()+
  theme(legend.position = "none")
```

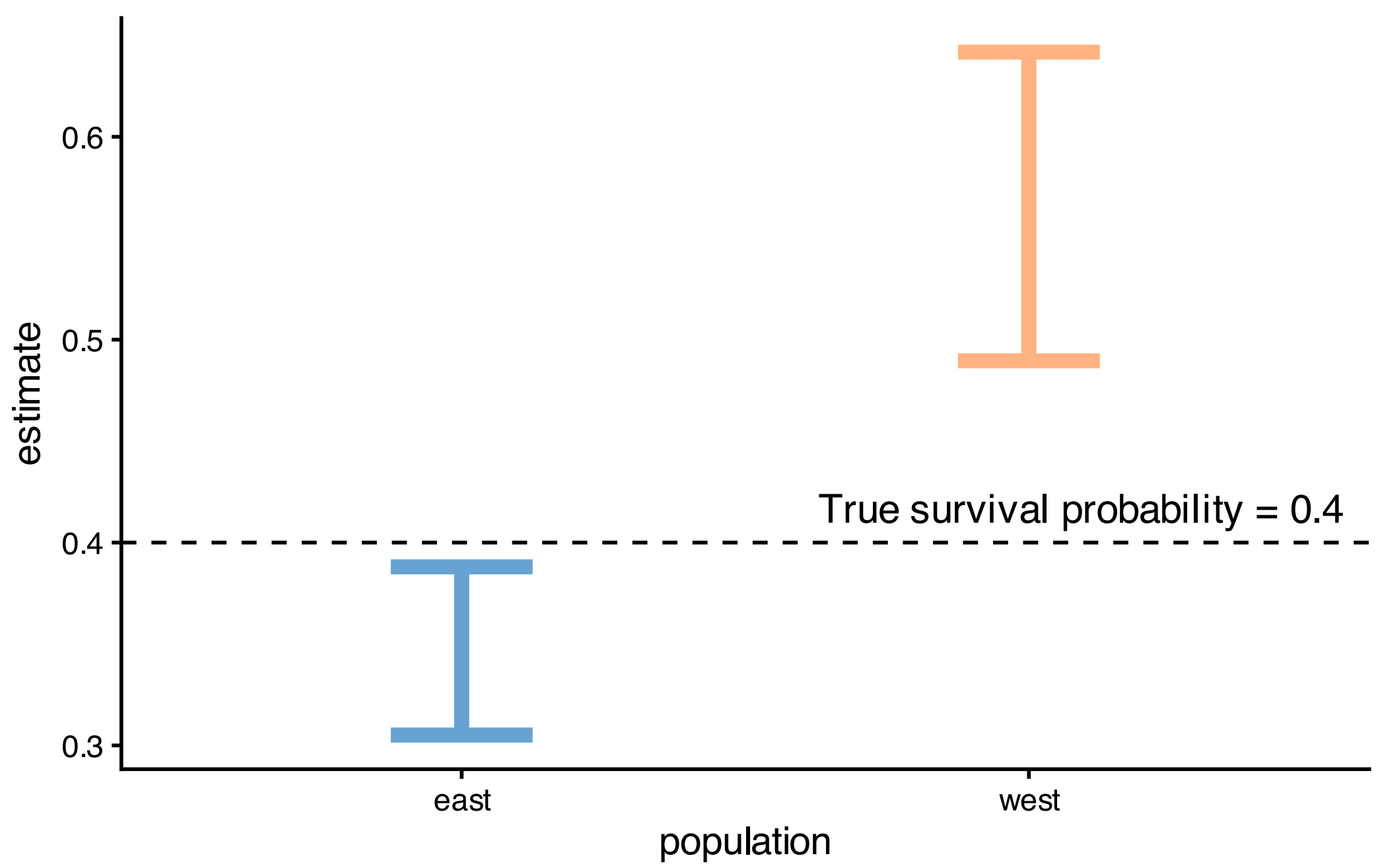

Conclusions

The simulations above, while simple, illustrate the potential inferential pitfalls researchers face when failing to account for the major violations of model assumptions attendent to automated telemetry data. Specifically, by failing to account for the several ways that availability for detection can vary among individuals, all the models above resulted in catastrophic inferential failures. nearly all parameters estimates were biased compared to the true survival probability. Moreover, each analysis inferred survival differences among groups that were entirely spurious, having only arisen due to group-specific differneces in availability for detection.
